# The differences of the precommissural and postcommissural fornix in the hippocampal location: a diffusion tensor tractography study

**DOI:** 10.1007/s00234-017-1817-z

**Published:** 2017-03-16

**Authors:** Sung Ho Jang, Sang Seok Yeo

**Affiliations:** 1 Department of Physical Medicine and Rehabilitation, College of Medicine, Yeungnam University, Daemyungdong, Namku, Daegu, 705-717 Republic of Korea; 2 Department of Physical Therapy, College of Health Science, Dankook University, 119, Dandae-ro, Dongnam-gu, Cheonan-si, Chungnam, 3116 Republic of Korea

**Keywords:** Diffusion tensor imaging, Precommissural fornix, Postcommissural fornix, Hippocampus, Anatomical location

## Abstract

**Purpose:**

The precommissural fornix and postcommissural fornix have different connections to the basal forebrain and septal region, and mammillary body, respectively. However, little is known about the differences of the precommissural fornix and postcommissural fornix in the hippocampal location. In this study, using diffusion tensor tractography, we investigated the differences of the precommissural fornix and postcommissural fornix in the hippocampal location.

**Methods:**

We recruited 25 healthy volunteers for this study. For reconstruction of the precommissural fornix and postcommissural fornix, we placed the seed region of interest on the septal nucleus, and the mammillary body, respectively. The target regions of interest (ROI) was given on the crus of the fornix on the coronal image. Evaluations of the anatomical location of the precommissural fornix and postcommissural fornix were performed using the highest probabilistic location in the hippocampal formation.

**Results:**

The precommissural fornix and postcommissural fornix were located at an average of 83.9 and 87.5% between the lateral margin of the red nucleus and collateral sulcus on the axial plane, and 77.2 and 81.4% between the lateral margin of the midbrain and the inferior longitudinal fasciculus on the coronal plane. Significant differences of location in the medio-lateral direction were observed in the axial and coronal plane (*p* < 0.05). However, no significant differences of location in the antero-posterior direction were observed between precommissrual and postcommissural fornix (*p* > 0.05).

**Conclusions:**

The reconstructed precommissural fornix and postcommissural fornix were connected to the cornu ammonis 1(CA1) of the hippocampus, and the precommissural fornix was located more laterally to the postcommissural fornix in the CA1.

## Introduction

The fornix is one of the principal fiber tracts providing the major afferent and efferent systems of the hippocampal formation [1, 2]. The precommissural fornix is mainly connected to the cholinergic nuclei in the basal forebrain and septal region from the hippocampal formation, and functionally contributes to central regulation of emotional behavior, motivation processes, and memory function [1–7]. The postcommissural fornix, on the other hand, is mainly concerned with transfer of information on episodic memory between the hippocampal formation and mammillary body [1, 2, 6, 7]. These differences of the precommissural fornix and postcommissural fornix in terms of anatomy and function suggest a possibility of different connections in the hippocampal subfields. However, little is known about the differences of the precommissural fornix and postcommissural fornix in the hippocampal subfields.

The recent development of diffusion tensor tractography (DTT), which is derived from diffusion tensor imaging (DTI), has enabled visualization and localization of the precommissural fornix and postcommissural fornix [2, 8–14]. Many studies have reported on injury of these neural tracts in various brain pathologies [8, 9, 13–19]. However, no study on the differences in anatomical location of the precommissural fornix and postcommissural fornix in the hippocampal location using DTT has been reported so far.

In the current study, using DTT, we attempted to identify the differences of the precommissural and postcommissural fornical fibers in the hippocampal location of normal subjects.

## Materials and methods

### Subjects

Twenty-five normal healthy subjects (14 males, 11 females; mean age, 31.12 ± 9.17 years; range, 20–49) with no history of neurologic disease were recruited for the study. All participants provided written consent prior to participation in the study, which was approved by the institutional review board at our hospital.

### Diffusion tensor image tractography

DTI data were acquired using a 6-channel head coil on a 1.5 T Philips Gyroscan Intera (Philips, Ltd., Best, The Netherlands) with single-shot echo-planar imaging. For each of the 32 non-collinear diffusion sensitizing gradients, we acquired 67 contiguous slices parallel to the anterior commissure-posterior commissure line. Imaging parameters were as follows: acquisition matrix =96 × 96, reconstructed to matrix =128 × 128 matrix, field of view =221 × 221 mm^2^, TR =10,726 ms, TE =76 ms, parallel imaging reduction factor (SENSE factor) =2, EPI factor =49 and *b* = 1000s/mm^2^, NEX =1, and a slice thickness of 2.3 mm (acquired isotropic voxel size 2.3 × 2.3 × 2.3 mm^3^).

### Fiber tracking

Diffusion-weighted imaging data were analyzed using the Oxford Centre for Functional Magnetic Resonance Imaging of the Brain (FMRIB) Software Library (FSL; www.fmrib.ox.ac.uk/fsl). Head motion effect and image distortion due to eddy current were corrected by affine multi-scale two-dimensional registration. Fiber tracking was performed using a probabilistic tractography method based on a multifiber model, and applied in the current study utilizing tractography routines implemented in FMRIB Diffusion (5000 streamline samples, 0.5 mm step lengths, curvature thresholds =0.2) [20]. The precommissural fornix and the postcommissural fornix were determined by selection of fibers passing through seed and target regions of interest (ROI). For analysis of the precommissural fornix, we placed the seed ROI on the septal nucleus on the coronal image, and the target ROI was given on the crus of the fornix on the coronal image [2]. For analysis of the postcommissural fornix, we placed the seed ROI on the mammillary body on the axial image, and the target ROI was given on the crus of the fornix on the coronal image [2].

### Measurements of anatomical location of the fornix at the hippocampus

**Fig. 1:**
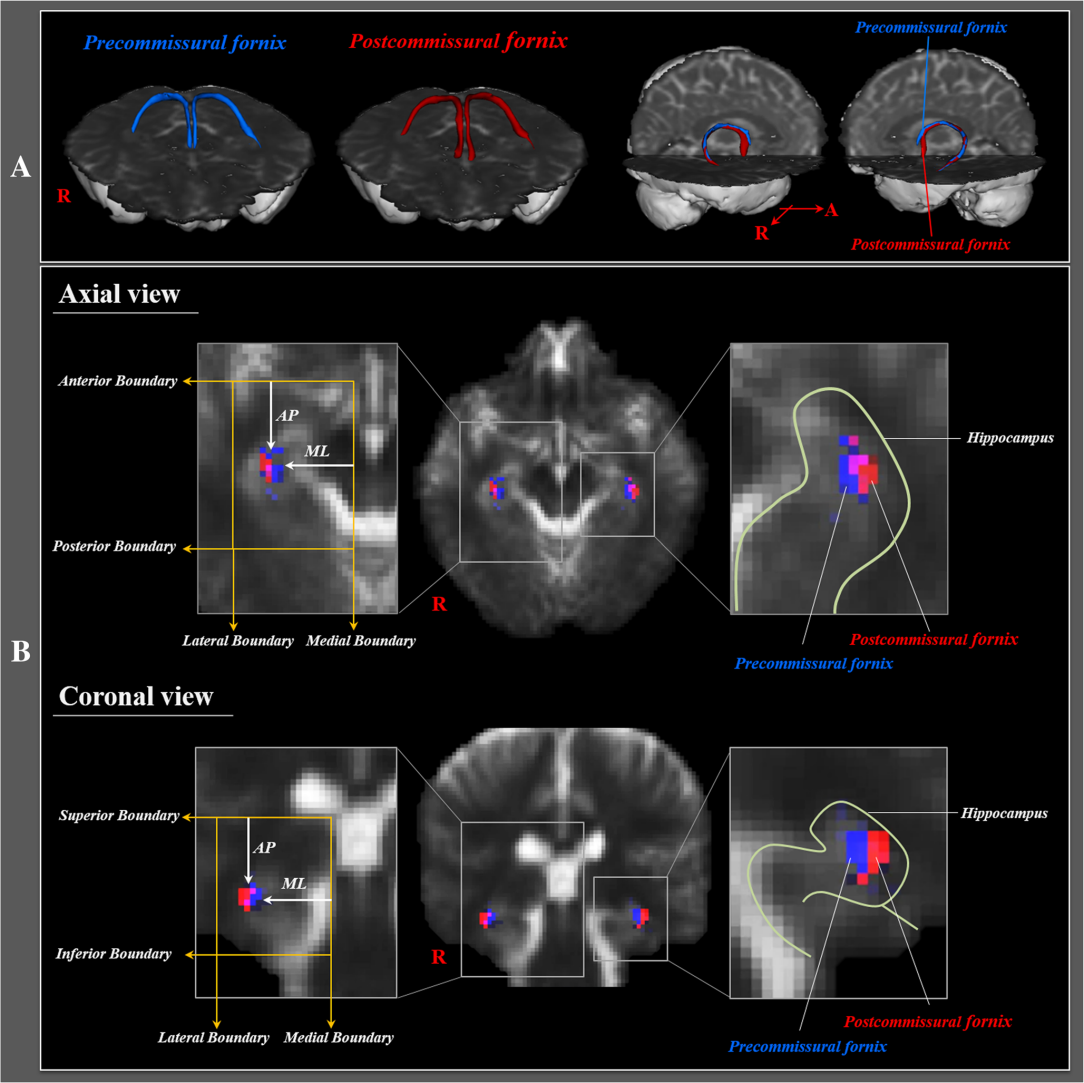
**a** Precommissural fornix (*blue*) and postcommissural fornix (*red*) were constructed in both hemispheres. **b** Landmarks used to determine the locations of the precommissural fornix and postcommissural fornix as follows: axial view: anterior boundary—hypothalamus, posterior boundary—the anterior margin of vermis of the cerebellum, medial boundary—the lateral margin of the red nucleus, lateral boundary—collateral sulcus; coronal view: superior boundary—the inferior margin of the lateral ventricle, inferior boundary—the inferior margin of the medial temporal lobe, medial boundary—the lateral margin of the midbrain, lateral boundary—inferior longitudinal fasciculus. The anatomical location of the fornix was measured laterally from the medial boundary in the medio-lateral direction (ML), and posteriorly from the anterior boundary in the antero-posterior direction (AP)

The anatomical location of the precommissural fornix and postcommissural fornix at the hippocampus was evaluated as the highest probabilistic location. As shown in Fig. 1, we defined the boundary as follows: axial view: anterior boundary—hypothalamus, posterior boundary—the anterior margin of vermis of the cerebellum, medial boundary—the lateral margin of the red nucleus, lateral boundary—collateral sulcus; coronal view: superior boundary—the inferior margin of the lateral ventricle, inferior boundary—the inferior margin of the medial temporal lobe, medial boundary—the lateral margin of the midbrain, lateral boundary—inferior longitudinal fasciculus. The anatomical location of the fornix was measured laterally from the medial boundary in the medio-lateral direction, and posteriorly from the anterior boundary in the antero-posterior direction (Fig. 1b).

The precommissural fornix and postcommissural fornix probabilistic maps were obtained by overlapping of the anatomical location of the highest probabilistic. Using SPM8 software (Wellcome Department of Cognitive Neurology, London, UK), the probabilistic map was superimposed on a mean non-diffusion-weighted image (b0), which was created using the mean of non-diffusion-weighted images of all subjects. In addition, non-diffusion-weighted images were normalized to the Montreal Neurological Institute (MNI) T2 template supplied with the SPM8 software.

### Statistical analysis

Data analysis was performed using SPSS software (v.15.0; SPSS, Chicago, IL, USA). Independent *t* test was used to determine difference of anatomical locations between precommissural fornix and postcommissural fornix on the hippocampus. Results were considered significant when *p* value was <0.05.

## Results

In all subjects, the precommissural fornix and postcommissural fornix originated from the hippocampal formation in each hemisphere as crus and both crus from the precommissural fornix and postcommissural fornix were joined to form the body of the fornix (Fig. 1a). The body of the fornix divided into each column of the fornix; each column of the precommissural fornix descended anteriorly to the anterior commissure and connected to the septal region and basal forebrain; in contrast, each column of the postcommissural fornix passed through the posterior to the anterior commissure, and connected to the mammillary body.

A summary of the average anatomical locations of the precommissural fornix and postcommissural fornix at level of the hippocampus on the axial and coronal plane is shown in Table 1. In the medio-lateral direction, the precommissural fornix was located at an average of 83.9% from the medial boundary of the axial plane and 77.2% on the coronal plane. By contrast, the postcommissural fornix was located at an average of 87.5% from the medial boundary of the axial plane and 81.4% on the coronal plane. In addition, significant differences of locations in the medio-lateral direction were observed between the precommissural fornix and postcommissural fornix (*p* < 0.05). In the antero-posterior direction, the precommissural fornix and postcommissural fornix were located an average of 42.5 and 41.7% from the anterior boundary at the axial plane, 45.9 and 46.9% at the coronal plane, respectively. However, no significant differences of locations in the antero-posterior direction were observed between the precommissural fornix and postcommissural fornix (*p* > 0.05).

**Table 1:** Average anatomical locations of the highest probability point of the precommissural fornix and postcommissural fornix in the hippocampal formation

	Direction	Right hemisphere		Left hemisphere		Total		*p*
		Pre-fornix	Post-fornix	Pre-fornix	Post-fornix	Pre-fornix	Post-fornix	
Axial plane	Medio-lateral	83.1 (4.9)	86.7 (4.5)	84.7 (6.6)	88.8 (5.4)	83.9 (5.8)	87.5 (5.0)	0.022*
	Antero-posterior	43.7 (9.5)	42.0 (11.2)	41.3 (14.4)	41.4 (11.6)	42.5 (12.1)	41.7 (11.3)	0.754
Coronal plane	Medio-lateral	77.9 (8.1)	81.2 (6.4)	76.6 (8.2)	81.7 (7.2)	77.2 (8.1)	81.4 (6.7)	0.021*
	Antero-posterior	47.0 (6.4)	47.8 (6.1)	44.9 (7.9)	45.9 (6.1)	45.9 (7.2)	46.9 (6.1)	0.546

Independent *t* test was used for determination of difference in anatomical location between the precommissural fornix and the postcommissural fornix

Values represent mean (±standard deviation), location (%)

*p < 0.05

## Discussion

In the current study, using DTT, we reconstructed the precommissural fornix and postcommissural fornix in the brain of normal subjects. The precommissural fornix descended anteriorly to the anterior commissure and connected to the septal region and basal forebrain. By contrast, the postcommissural fornix passed posteriorly to the anterior commissure and connected to the mammillary body. Based on the reconstructed fornix, we investigated the anatomical location of the precommissural fornix and postcommissural fornix in the hippocampus and found the following results: all reconstructed precommissural fornix and postcommissural fornix were mainly connected to the cornu ammonis 1(CA1), which is the first region in the hippocampal circuit. However, the anatomical location in the CA1 was different: in detail, the precommissural fornix was located more laterally to the postcommissural fornix in the CA1; in contrast, the precommissural fornix and postcommissural fornix did not show difference in the antero-posterior direction.

Using surface anatomy or neuroimaging techniques, many studies have reported on the anatomical characteristics of the precommissural fornix and postcommissural fornix in the animal and human brain [2–7]. In particular, some animal studies reported on the anatomical locations of the fornix on the hippocampal region, and subiculum and CA1 of hippocampal subfields are known to be the principal source of efferent fibers for the fornix [21, 22]. However, little is known about the detailed anatomical information on the precommisural and postcommissural fornix on the hippocampus in the human brain. On the other hand, even without exact estimation of the anatomical location of the fornix on the hippocampal region, several studies have demonstrated functional correlation between fornix and hippocampal formation [17, 18, 23]. In 2011, using postmortem MRI and histopathology, Dawe et al. found hippocampal deformations by Alzheimer’s disease commonly observed in the CA1 subregion and subiculum of the hippocampus without change of CA2 and 3 subregions; these deformations have shown significant correlation with decrease of episodic memory, semantic memory, and working memory [17]. In 2012, using DTI, Lee et al. reported significant association of memory problems of patients with mild cognitive impairment and Alzheimer’s disease with the reduction of hippocampal CA1 area. In addition, they also suggested significant correlation of the reduced volume of the hippocampal CA1 area with decreased value of fractional anisotropy of the fornix [23]. In a recent study, using DTI, Fletcher el al suggested that the volume of the fornix and axial diffusivity can be used for predictors of cognitive decline in normal elderly persons, and they also demonstrated a strong association of the thickness of the hippocampal CA1 area with volume of the fornix [18]. Although these previous studies could not show the difference for the anatomical locations of the precommissural fornix and postcommissural fornix in the hippocampal subfields, these results appear to be compatible with the results of the current study, which showed connection between the precommissural fornix and postcommissural fornix and hippocampal CA1 area. As a result, to the best of our knowledge, this is the first study using DTT to investigate the differences of the precommissural fornix and postcommissural fornix in the hippocampal location in the human brain. However, the limitations of DTI should be considered. In particular, regions of fiber complexity and fiber crossing could prevent full depiction of the underlying fiber architecture on DTI [19, 24, 25]. In addition, we could not classify the exact pathway of the precommissural fornix and postcommissural fornix in the body and crus of fornix.

In conclusion, we found the difference of the precommissural fornix and postcommissural fornix in the hippocampal location in normal subjects: both fornices were mainly connected to the hippocampal CA1 area, and the precommissural fornix was located on the more lateral portion of the CA1 area, compared with the postcommissural fornix. Conduct of further studies on the functional difference according to the anatomical difference of the precommissural fornix and postcommissural fornix should be encouraged. In addition, clinical significance of these differences in patients with brain injury should be elucidated.
